# Quantitative Stratification of Diffuse Parenchymal Lung Diseases

**DOI:** 10.1371/journal.pone.0093229

**Published:** 2014-03-27

**Authors:** Sushravya Raghunath, Srinivasan Rajagopalan, Ronald A. Karwoski, Fabien Maldonado, Tobias Peikert, Teng Moua, Jay H. Ryu, Brian J. Bartholmai, Richard A. Robb

**Affiliations:** 1 Department of Physiology and Biomedical Engineering, Mayo Clinic, Rochester, Minnesota, United States of America; 2 Division of Pulmonary and Critical Care Medicine, Mayo Clinic, Rochester, Minnesota, United States of America; 3 Department of Radiology, Mayo Clinic, Rochester, Minnesota, United States of America; University of Tübingen, Germany

## Abstract

Diffuse parenchymal lung diseases (DPLDs) are characterized by widespread pathological changes within the pulmonary tissue that impair the elasticity and gas exchange properties of the lungs. Clinical-radiological diagnosis of these diseases remains challenging and their clinical course is characterized by variable disease progression. These challenges have hindered the introduction of robust objective biomarkers for patient-specific prediction based on specific phenotypes in clinical practice for patients with DPLD. Therefore, strategies facilitating individualized clinical management, staging and identification of specific phenotypes linked to clinical disease outcomes or therapeutic responses are urgently needed. A classification schema consistently reflecting the radiological, clinical (lung function and clinical outcomes) and pathological features of a disease represents a critical need in modern pulmonary medicine. Herein, we report a quantitative stratification paradigm to identify subsets of DPLD patients with characteristic radiologic patterns in an unsupervised manner and demonstrate significant correlation of these self-organized disease groups with clinically accepted surrogate endpoints. The proposed consistent and reproducible technique could potentially transform diagnostic staging, clinical management and prognostication of DPLD patients as well as facilitate patient selection for clinical trials beyond the ability of current radiological tools. In addition, the sequential quantitative stratification of the type and extent of parenchymal process may allow standardized and objective monitoring of disease, early assessment of treatment response and mortality prediction for DPLD patients.

## Introduction

Diffuse parenchymal lung diseases (DPLDs) encompass a set of diseases characterized by widespread abnormalities of the lung parenchyma. Patients with DPLD represent a substantial healthcare burden due to high disease prevalence, chronic nature of such processes and lack of curative therapy with associated morbidity and premature mortality [1], [2]. Despite advances in modern medicine, including medical imaging, pathology and genomics, diagnosis and treatment of DPLD remain difficult. The American Thoracic Society / European Respiratory Society [3], [4] strongly recommends a multidisciplinary approach to diagnosis in DPLDs based on consensus of radiologists, clinicians and pathologists. Unfortunately, a successful classification scheme that allows recognition of disease groups or even consensus diagnosis of specific diseases identically across radiology, pulmonary and pathology disciplines remains ambiguous [5].

Individualized treatment strategies based on patient-specific disease manifestations are the ultimate goal of modern pulmonary medicine. In reality, this lofty goal is unachievable with our current medical knowledge. Obstacles include lack of established diagnostic, prognostic and predictive patient-specific biomarkers. Nevertheless, with present day technologies and innovative strategies the practice of stratified medicine is becoming more feasible. Patient populations can be stratified based on quantifiable characteristics that define the disease and characterize key physiologic, pathologic, anatomic or patient-reported factors that impact quality of life, morbidity and mortality. The goal of stratified medicine entails the ability to guide individualized patient care based on recognized disease characteristics and established prognostic features of matched group [6]. However, the definitions of disease phenotypes and prognostic and predictive biomarkers to guide patient management remain elusive for DPLD [7].

High-resolution computed tomography (HRCT) is the preferred radiologic imaging modality for evaluating lungs. In current clinical practice, HRCT adds tremendous value in its ability to diagnose and manage patients with DPLDs and may often obviate or direct specific targets for surgical lung biopsies [8]–[14]. It can be both diagnostic and prognostic for some pathological processes. Several CT-based automated quantitative methods (quantitative CT - QCT) have been proposed to quantify and characterize parenchymal abnormalities [15]–[19]. QCT has demonstrated correlation of quantified parenchymal patterns with well-accepted clinical endpoints – physiologic indices, visual radiology scores, as well as prognostic outcomes for subsets of diseases [16], [19]–[21]. Furthermore, correlation of quantified parenchymal abnormalities with pathologic features has resulted in confident interpretation and diagnosis of certain diseases (e.g., usual interstitial pneumonia) obviating the need for surgical lung biopsy [3]. The quantitative nature of radiological image-based biomarkers [22] enables development of an automated and consistent image-based methodology to achieve objective population stratification.

We stratified a population based on the hypothesis that the radiological distribution and extent of quantified parenchymal abnormalities, as quantified by QCT analytic tools, is characteristic of disease severity and subtype and successfully evaluated with the clinically accepted physiologic measurements, visual radiology scores and survival indices.

## Materials and Methods

### Data

The population data for this study was obtained from Lung Tissue Research Consortium (LTRC) database. LTRC is a NIH/NHLBI-sponsored, multi-site initiative with repository of clinical data, pathologic specimens, CT scans and QCT characterizations of ILD and COPD patients enrolled in the study. Radiology department at Mayo Clinic serves as the LTRC radiology core and consequently, the quantitative analysis of CT scans was performed using in-house software Computer-Aided Lung Informatics for Pathology Evaluation and Rating (CALIPER) [15], [16] developed at Mayo Clinic. The CT scans, clinical data, quantitative analysis and diagnosis information used in this study can be requested online at http://www.ltrcpublic.com/data_requests.htm.

#### (a) Population QCT

The input data for this study is the CALIPER-based quantification of parenchymal CT patterns. Briefly, CALIPER processes and characterizes the CT dataset by isolating the lung parenchyma and classifying every parenchymal voxel into one of the following characteristic CT patterns: normal (N), reticular (R), honeycomb (HC), ground-glass (GG), mild low attenuation areas (LAA), moderate LAA and severe LAA. The reliability of CALIPER-based classification of parenchymal patterns was evaluated for presence of artifacts or other image quality deficiencies such as respiratory motion or segmentation inaccuracies by a thoracic radiologist (BJB) as part of LTRC protocol. The efficacy of quantitative characterization of parenchymal patterns by CALIPER was ascertained and reported outside this study [15], [16].

The candidates for our study were selected retrospectively based on quality of CT scans. Scans performed at full inspiration at supine position from Siemens or GE scanners acquired with thin collimation width (1 mm to 2.5 mm) were our inclusion criteria for quantitative analysis. Further, candidates with scans performed with intravenous contrast, with significant artifacts (such as beam hardening due to metallic hardware, respiratory motion or other image quality errors) or reconstructed using sharp, edge enhancing algorithms such as B70 or B80 (Siemens) or Lung (GE) were excluded. The demographics and final diagnosis for the identified 1322 patients is outlined in **Table S1**.

#### (b) Clinical Variables

The clinical data retrieved for the identified patients included visual semi-quantitative radiology scores, physiologic measurements, BODE (**B**ody mass , airflow **O**bstruction, **D**yspnea, **E**xercise capacity) index [23], GOLD (**G**lobal initiative for chronic **O**bstructive **L**ung **D**isease) classification label [24] and **S**t. **G**eorge’s **R**espiratory **Q**uestionnaire (SGRQ) patient questionnaire scores [25]. The semi-quantitative radiology scores of visual abnormalities for each subject with CT data were coded from 0 to 4 to respectively represent 0% (none), 1% to 25% (mild), 26% to 50% (moderate), 51% to 75% (marked) and 76% to 100% (severe) of abnormality type in each of the 12 regions: the central and peripheral zones of the left and right upper, middle and lower lobes (lingual on the left). We combined the regional abnormality scores to get the score for the whole lung The physiologic measures extracted from the database included pulmonary function test (PFT) results comprising of forced expiratory volume in 1 second (FEV1), forced vital capacity (FVC), diffusing lung capacity (DLCO and total lung capacity (TLC). All the above mentioned scores, indices and labels were used to validate the clinical relevance of the stratified groups.

### Dissimilarity metric

Based on the hypothesis that the distribution of the CT patterns (N, R, HC, GG, mild LAA, moderate LAA and severe LAA) is a characteristic of the disease subtypes, a pairwise dissimilarity metric was designed and pairwise comparisons between the 1322 lungs were computed. The dissimilarity (D) between lungs A and B uses a combination of (i) dissimilarities in the global distribution of all the seven abnormalities between cases A and B; (ii) regional dissimilarities between A and B based on (a) an asymmetric weight - regional volume proportion relative to total volume of case A; (b) dissimilarities in the proportions of volumes in the corresponding regions of A and B; and (c) dissimilarity in the proportions of parenchymal patterns in each corresponding region in A and B.

### Equation 1:


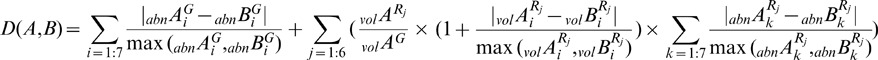
 where, 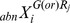 represents the percentage abnormality distribution of *i^th^* (of seven) CT pattern in the whole lung (G) (or) in *j^th^* (of six) region of the lung (*R_j_*). 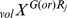
represents the total volume of the lung (G) (or) in *j^th^* region of the lung (*R_j_*). *X* represents a patient’s CT lung volume.

### Stratification method

Based on the above mentioned dissimilarity metric, 1322 cases are compared pairwise. The unique clusters representing similar groups of patients are identified by clustering the 1322x1322 dissimilarity matrix in a unsupervised manner using Affinity Propagation (AP) [26]. The salient feature of AP is that it does not require a priori specification of the number of desired clusters, unlike k-means and hierarchical clustering techniques where the number of desired clusters is required. AP uses message passing to identify exemplars and candidates representing naturally unique clusters. This is critical to identifying the unique radiologically similar groups of cases in the database in a truly unsupervised manner. However, AP uses a preference parameter to promote a set of candidates to be exemplars. To get an unbiased stratification, we assigned equal preferences to all the candidates. The preference was set to the median of negative squared values of dissimilarities. Such an initialization is known to yield many small clusters with highly similar data grouped together. We followed a two-pass approach to facilitate clinically meaningful clusters. The median preference AP-based cluster groups were identified in first pass. The clusters were further evaluated with **AN**alysis **O**f **SIM**ilarities (ANOSIM) [27], a statistical analysis method which performs iterative permutations to identify the separation between cluster groups by computing R values for every pair of clusters (R-value is close to 1 for dissimilar clusters and 0 for similar ones). The pairwise ANOSIM R values for the clusters from first pass were clustered using AP to arrive at the final clusters.

### Correlation of stratified groups with clinical variables

One-way analysis of variance (ANOVA) was used to assess the discriminability of the physiologic measures across the quantitatively stratified groups. FEV1-FVC ratio and percentage predicted values of DLCO, TLC, FEV1 and FVC were the considered physiologic measures. Post-ANOVA pairwise t-tests are performed. Bonferroni correction computed as 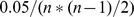
(*n*  =  number of groups) was applied to the p-value.

The clusters were identified as fibrotic and obstructive based on the glyphs within the individual clusters. This identification was done to perform further disease specific analysis. For populations within fibrotic clusters, the GAP-based one-year mortality predictions [28] were computed statistically compared across groups using student’s t-test. Similar test for performed for populations in obstructive clusters based on BODE index.

GOLD classifications for patients in obstructive clusters were retrieved from LTRC database. LTRC uses the older GOLD classification system wherein for patients with FEV1-FVC ratio less than 0.7; the FEV1% predicted values are used to stage the disease 1 through 4 to respectively categorize disease into mild, marked, severe and very severe forms. We also computed the GOLD labels using the new GOLD classification [29]. This new classification based on FEV1% predicted values and modified Medical Research Council (mMRC) dyspnea score groups patients as A, B, C and D to define patients with low risk-less symptoms, low risk-high symptoms, high risk-less symptoms and high risk-high symptoms, respectively.

## Results

### Quantitative Characterization and Representation

**Figure 1:**
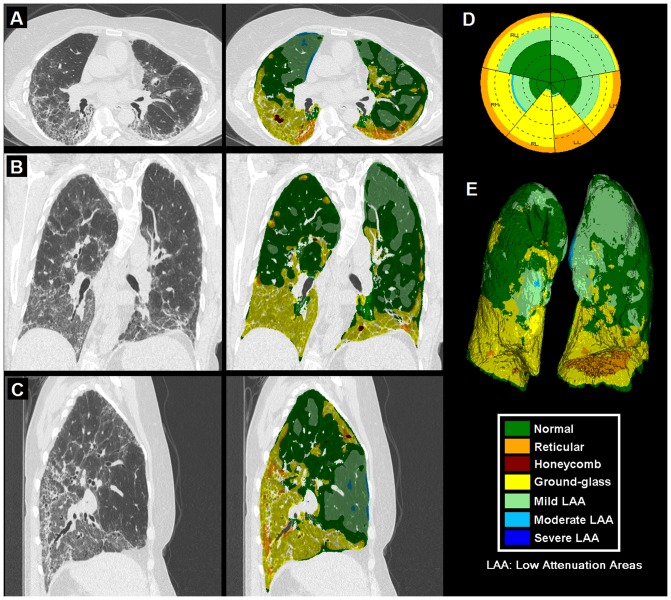
Representative CT lung volume characterized into parenchymal patterns by Computer Aided Lung Informatics for Pathology Evaluation and Rating (CALIPER). It illustrates colored overlay in axial (A), coronal (B) and sagittal (C) sections with glyph (D) and 3D rendering (E). The color key for the seven patterns is also shown.

**Figure 2:**
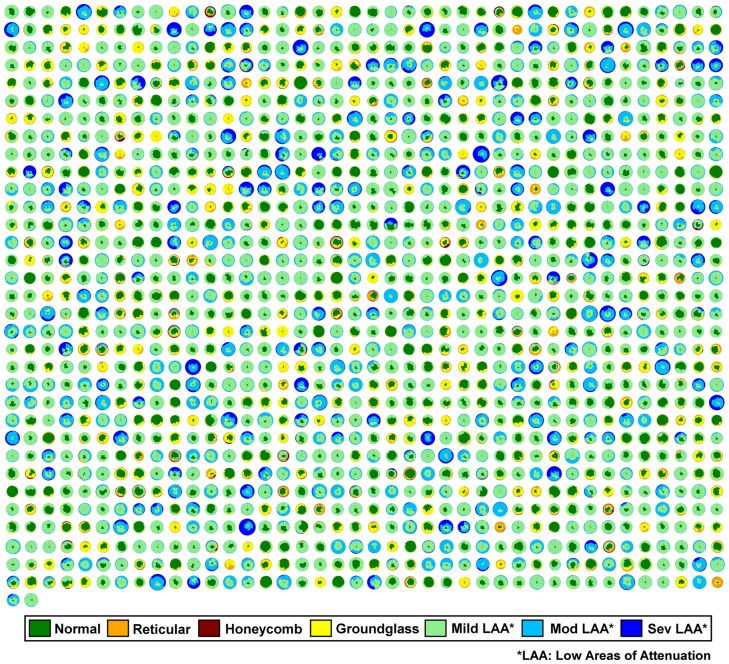
1322 LTRC patients represented as glyphs. The classified parenchymal patterns are represented in the indicated colors. The radius of the glyphs is proportional to the lung volumes; the COPD cases with extensive low attenuation areas likely due to emphysema are visually larger than more normal or fibrotic cases with considerable regions of reticular or honeycombing.

**Figure 1** shows a representative dataset with axial, coronal and sagittal sections of a CT lung volume where every voxel of the parenchyma is characterized and color coded into one of the parenchymal patterns (N, R, H, G, and mild, moderate and severe LAA). The overall distribution is illustrated using glyph representation [15], [30] and 3D rendering. Briefly, the glyph illustrates the regional composition of classified lung volume. The origin of the glyph was fixed at 12 o’clock starting with the left upper lobe. The individual regions span through angles proportional to their respective volumes. Within each region, the abnormality distribution was represented by color-coded sectors proportional to the percentage of abnormality in the region. The concentric circles were drawn at 20% intervals for enhanced visualization. **Figure 2** shows glyph representations of all the 1322 patients used in the study. The radius of the individual glyphs is proportional to the patient’s lung volumes. The illustration provides a visual summary of the disease spectrum in the database. Cases of parenchymal fibrosis and obstructive disease such as emphysema can be visually differentiated based on the size of each glyph (proportional to lung volumes) and composition. Opacities characteristic of fibrotic processes such as G (yellow), R (orange) or H (red) are readily apparent, as are low attenuation areas characteristic of hyperinflation or various severity of parenchymal destruction due to emphysema or bullae (light green, light blue or dark blue).

### Quantitative Stratification

**Figure 3:**
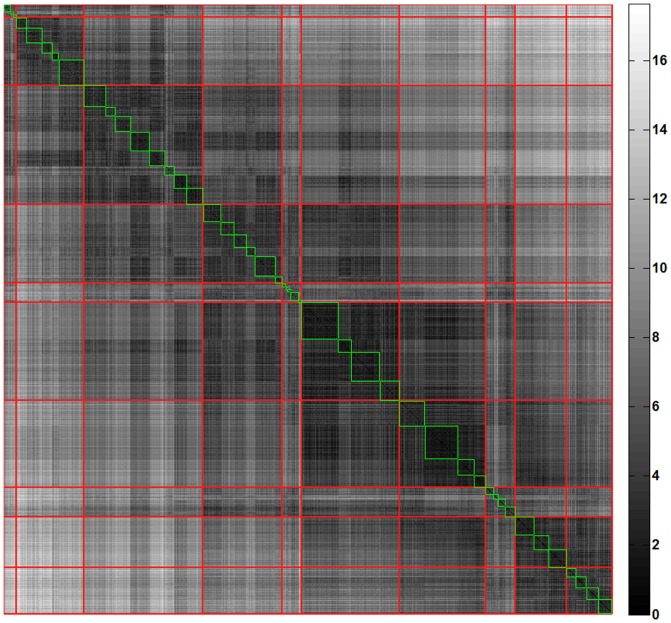
The permuted dissimilarity matrix (1322×1322) representing the abnormality based pairwise dissimilarities (brighter the shade higher the dissimilarity). The green blocks illustrate the first-pass clusters and the red diagonal blocks represent the final ten stratified clusters.

**Figure 4:**
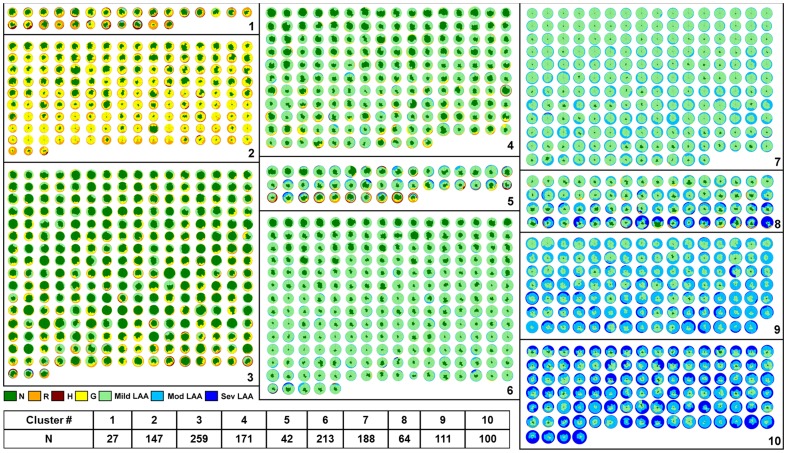
The ten stratified groups of 1322 patients represented as glyphs. The groups were the result of quantitative unsupervised clustering based on dissimilarity metric that captures the distribution of classified parenchymal patterns.

The distribution and location of parenchymal abnormalities in the lungs are central to CT-based diagnosis as they are indicative of disease type (diagnosis) and severity [3]. The quantitative measure of dissimilarities between pairs of patients based on such distribution is computed using the dissimilarity metric to further stratify similar patient cohort. The first-pass of stratification based on unsupervised clustering, AP, yielded 46 groups of patients with highly similar radiological distribution of the seven classified patterns. In the second pass, 46 clusters were merged further using AP of cluster wise ANOSIM R values resulting in the final 10 unique clusters. **Figure 3** illustrates the pairwise dissimilarity matrix representing the first pass (blocks in green) and second pass (blocks in red) clusters. **Figure 4** represents the glyph visualizations of 1322 patients grouped into the ten identified clusters. Based on visual inspection of the glyphs within the respective clusters, the clusters were ordered from most fibrotic (cluster 1) to most extensive changes from COPD, with low attenuation areas (cluster 10). Clusters (1, 2, and 3) and clusters (6, 7, 8, 9, and 10) in **Figure 4** were visually identified as fibrotic and obstructive patient clusters, respectively, for disease-specific analyses described in the following sections.

### Correlation of Quantitative Stratification with Clinical indices

The stratified groups were obtained solely based on CALIPER-quantified radiological characteristics blinded to any clinical or physiologic information. The following subsections show discriminability of the clinical variables across the ten identified clusters of patient population.

#### (a) Relationship with semi-quantitative visual radiology scores

**Figure 5:**
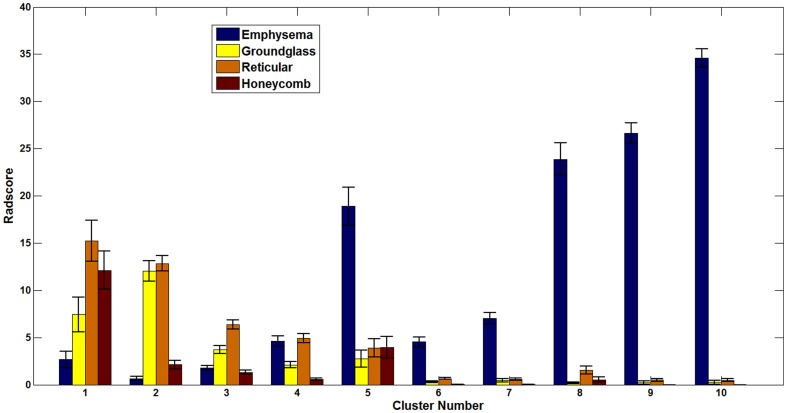
The plot shows the mean population values of the semi-quantitative visual radiology scores (assigned by LTRC radiologist) for each parenchymal abnormality in each cluster. The error bar is the standard error of mean of scores across the patients in the cluster.

Visual radiology scores are generally used to validate the efficacy of automated methods. The semi-quantitative visual score for each patient which is the aggregate of regional scores for emphysema, reticular infiltrates, ground-glass and honeycomb parenchymal patterns were retrieved from the LTRC database for the 1322 patients. The emphysema pattern on CT scans is evaluated based on lower density regions, which are characterized by CALIPER as mild, moderate and severe forms of LAA. **Figure 5** illustrates the cluster-specific mean scores for each pattern across the population in each cluster. The prominent visual radiology scores for fibrotic patterns (ground-glass, reticular and honeycombing) in the first three clusters and emphysema patterns in the last five clusters can be visually validated from the glyphs within each group (**Figure 4**). Additionally, visual radiology scores in cluster 5 revealed mixed fibrotic and emphysematous diseases which were independently reflected in the glyphs specific to that cluster as shown in **Figure 4**.

#### (b) Relationship with physiologic measures

**Figure 6:**
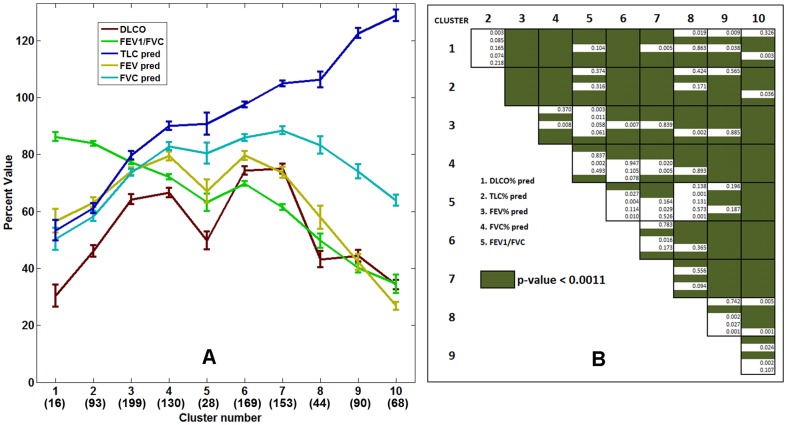
The cluster-specific mean physiologic measures (FEV1/FVC ratio, percentage predicted values of DLCO, TLC, FEV1, and FVC) for the ten stratified groups. The error bars indicate the standard errors of mean. The numbers within parenthesis in the horizontal axis represent the number of cluster-specific cases used in the mean, standard error computation. The post-ANOVA pairwise t-test for the indicated five variables is shown as the staircase diagram with green fill for significant differences after Bonferroni correction. At least one variable is statistically significant across the clusters except for between groups (1, 2) and (5, 8).

Physiologic measures are considered the clinical biomarkers for the functional status and assessment of disease progression in patients with DPLD and represent generally accepted endpoints in clinical trials [31], [32]. They include pulmonary function tests (PFTs) which measure forced vital capacity (FVC), forced expiratory volume in 1 second (FEV1), total lung capacity (TLC) and FEV1/FVC ratio. The cluster-specific means and standard errors of mean of these measures are shown in **Figure 6A**. Statistically significant differences in the distribution of the physiologic measures were noted based on one-way ANOVA analysis performed on each physiologic variable across the clusters (p-value < 0.0001 in each case). **Figure 6B** shows the results of post-ANOVA pairwise t-tests. At least one variable was found statistically significant (Bonferroni corrected p-value < 0.0011) across multiple comparisons except for cluster pairs (1, 2) and (5, 8). Clusters 1 and 2 are both severe fibrotic groups with different distribution of honeycomb and reticular patterns and therefore portray similarly impaired pulmonary function characteristics. Cluster 5 represented concurrent fibrosis and mild to moderate forms of LAA representing emphysema. Cluster 8 was composed primarily of severe LAA with minimal fibrosis in the bases for a portion of cases, with similar physiologic measures as those in cluster 5. The highest FEV1/FVC ratio and the lowest TLC in cluster 1 and least FEV1/FVC ratio and highest TLC in cluster 10 were reflective of known clinical characteristics of severe fibrotic and emphysematous disease, respectively [33]. Furthermore, the sudden drop in DLCO for group 5, with radiologically mixed fibrosis and obstructive characteristics, is in agreement with findings commonly seen in combined pulmonary fibrosis and emphysema (CPFE) disease [34].

#### (c) Relationship with GAP, GOLD, BODE and SGRQ scores

**Figure 7:**
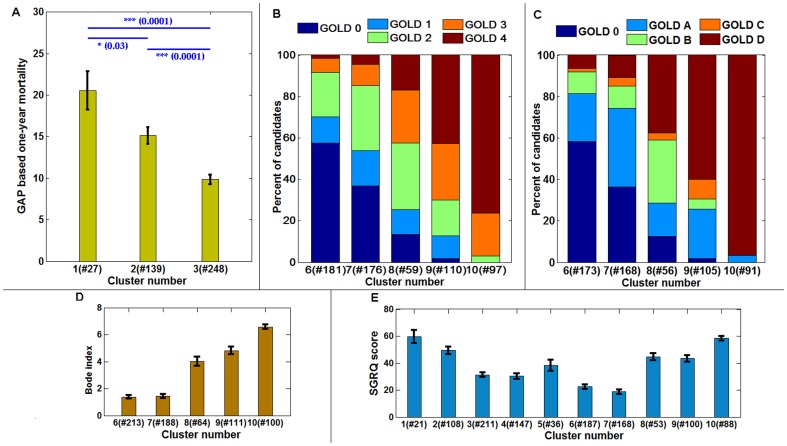
The correspondence of fibrotic and obstructive clusters with established indices and disease classification. The Gender, Age and Physiology (GAP) based one-year mortality predictions for clusters 1, 2 and 3 in Figure 3 (A). The distribution of the old GOLD category (B) and new GOLD category (C) distribution across the obstructive clusters: 6, 7, 8, 9 and 10. The mean distribution of BODE indices across the obstructive clusters (D) and, the mean score distribution of SGRQ patient questionnaire scores of patients across all the clusters (E). The number of samples available in each cluster is noted in the horizontal axis. The error bars indicate the standard error of mean.

GAP based prediction of survival in patients with idiopathic pulmonary fibrosis (IPF) is a validated index and staging system proposed to enable differential effect in disease management [28]. Based on GAP index, one-year mortality predictions were computed for patients in fibrotic clusters (1, 2 and 3). **Figure 7A** shows the statistically significant differences in the mean of the one-year mortality estimates of the patients across the three clusters (p-value  =  0.03 between clusters 1 and 2; p-value  =  0.0001 between clusters 2 and 3 as well as clusters 1 and 3).

GOLD classification guidelines [24] categorize patients into four stages based on FEV1% predicted values to assess disease severity and direct disease management. The GOLD criteria for the patients in the obstructive clusters (6, 7, 8, 9 and 10) were retrieved from LTRC database. **Figure 7B** shows the distribution of patients with GOLD scoring (1: mild; 2: moderate; 3: severe and 4: very severe). To overcome inefficiencies in the old classification guideline [29], a new GOLD classification scheme which incorporates dyspnea score with FEV1% predicted values has been recently proposed to better assess and predict exacerbations among COPD patients. The new GOLD classification uses categories A, B, C and D, respectively for patients with low risk-low symptom, low risk-high symptom, high risk-low symptom and high risk-high symptom. **Figure 7C** shows the composition of the new GOLD classifications across the five quantitatively derived obstructive clusters. For cluster 10 all patients in GOLD stages 3 and 4 (according to the old classification) fell into the new GOLD category D with expected higher severity of disease burden and incidence of exacerbations. Quantitatively stratified groups with emphysematous parenchyma appear to correlate with disease severity as measured by radiology and clinical GOLD classifications.

**Figure 7D** shows the trend in BODE index for obstructive clusters with significantly different range of BODE scores across all the cluster pairs except (6, 7) and (8, 9). **Figure 7E** shows the trend of SGRQ scores respectively across the clusters. For both BODE and SGRQ scores, higher scores reflect worse health condition. Although, BODE is generally used to assess COPD patients, the distribution across all ten clusters showed similar trend as SGRQ scores (**Figure S1**). Similarly, trend in GAP-based one-year mortality predictions are similar to BODE and SGRQ for the first three clusters (**Figure 7** **and Figure S1**). It is interesting to note that cluster 5, representing the mixed obstructive and restrictive abnormality, has considerably high scores of BODE and SGRQ compared to the neighbor clusters, although physiologic measures (in **Figure 6**, except DLCO) were in a continuum.

## Discussion

We have developed an automated and unsupervised method to quantitatively stratify the patient populations from the LTRC database. The ten groups exhibit unique clinically relevant distributions of lung function measurements. Furthermore, the obstructive and fibrotic groups show correlation with GOLD criteria and survival predictions based on GAP, respectively. The BODE scores, generally adopted biomarker index for COPD patients, show significant differences across obstructive groups. The efficacy of the stratification is visually captured through a succinct glyph-based representation of the multi-variable image data. **Figure 4** represents about 200 gigabytes of image data from 1322 cases organized into subpopulation groups of similar radiological features where each patient lung is further characterized into six regions and seven characteristic parenchymal pattern proportions. Glyph-based visualization offers visual interpretation of the analytic process and representation of results, which would otherwise be impossible by comparing 600,000 image slices or numerical representation of regional characteristics across 1322 cases.

A fundamental problem in the diagnosis, severity assessment, individualized management and outcome analysis for DPLD is the lack of a robust and objective biomarker [35]. Although HRCT is integral to clinical diagnosis and management of DPLDs, its use in clinical trials is sparse [36]. The opportunities of QCT as a viable biomarker is being explored [35] and several techniques for characterization of lung parenchymal disease with validated correlations of classified parenchymal patterns with physiology, visual radiology scores and patient survival have been proposed [16], [19], [37], [38]. However, a recent editorial summarizes the present situation, “*it is technically challenging to efficiently extract information on these patterns from CT scans … and there still seems to be a long way to go before computer software can automatically detect distinct and intuitively meaningful phenotypes*” [39]. Additionally, the challenges involved in optimization and standardization of acquisition and reconstruction protocols has limited the use of CT / QCT in multi-center clinical trials [35], [36]. The LTRC CT scans were acquired in four different centers and therefore involved use of a range of acquisition and reconstruction kernels. The CALIPER characterization of lung parenchyma and quantitative stratification reported in this paper was ascertained to be consistent and reproducible across several reconstruction protocols. **Figure S2** shows glyph illustrations of different data reconstructions for two representative LTRC cases. Cases (A) and (B) represent respectively fibrotic and obstructive parenchymal abnormalities. The variations in the glyphs across the reconstruction settings are minimal. The proposed method consistently categorized all the reconstructions of cases (A) and (B) into cluster 3 and cluster 10, respectively (detailed description is provided in File S1).

The study reported in this paper could be strengthened using an independent, even smaller DPLD population. LTRC study does not have patient follow-up and consequently, the efficacy of quantitative stratification could not be assessed with survival outcome. The methodology could also be investigated to investigate the stratification effects in response to an intervention or longitudinal disease progression. Notwithstanding the aforementioned limitations, the proposed stratification methodology can be extended to sub-stratify and identify radiological heterogeneity within the grouped population. This could be useful to assess the radiological phenotypes and possibly different prognostic and therapeutic implications in patients. There is a need for reliable and sensitive measures to evaluate clinical significance and track efficacy of treatments in clinical trial cohorts [32]. The CT-based quantitative stratification could be an objective step to address this unmet need. We believe that, with further validation, meaningful information can be objectively interpreted based on the proposed quantitative stratification tool, *just-in-time* automated quantification software such as CALIPER and efficient glyph-based visualization. This can enable futuristic objective of *physician-in-the-loop* interpretation and evaluation of lung parenchymal disease that can reduce technical burden to the end user and facilitate clinical translation.

## Supporting Information

Figure S1 The mean distribution of the BODE indices across the ten clusters. The error bars represent standard error of mean. The BODE is generally defined for obstructive cluster and Figure 7D illustrates the distribution for clusters 6 through 10. The trend in BODE distribution across clusters resemble the SGRQ (Figure 7E).
(TIF)

Figure S2 **Glyph representations of multiple reconstructions of two LTRC patients.** All the data reconstructions for both cases, (A) and (B) were consistently categorized into cluster 3 and cluster 10, respectively.
(TIF)

Table S1 **Patient demographics and major diagnosis of LTRC cohort.**
(DOCX)

File S1 (DOCX)

## References

[1] Olson AL, Swigris JJ, Lezotte DC, Norris JM, Wilson CG, et al (2007) Mortality from pulmonary fibrosis increased in the United States from 1992 to 2003. American Journal of Respiratory and Critical Care Medicine 176: 277–284. 17478620 10.1164/rccm.200701-044OC

[2] Mannino DM, Buist AS (2007) Global burden of COPD: risk factors, prevalence, and future trends. Lancet 370: 765–773. 17765526 10.1016/S0140-6736(07)61380-4

[3] Raghu G, Collard HR, Egan JJ, Martinez FJ, Behr J, et al (2011) An official ATS/ERS/JRS/ALAT statement: idiopathic pulmonary fibrosis: evidence-based guidelines for diagnosis and management. Am J Respir Crit Care Med 183: 788–824. 21471066 10.1164/rccm.2009-040GL PMC5450933

[4] Travis WD, Costabel U, Hansell DM, King TE Jr, Lynch DA, et al (2013) An official American Thoracic Society/European Respiratory Society statement: Update of the international multidisciplinary classification of the idiopathic interstitial pneumonias. Am J Respir Crit Care Med 188: 733–748. 24032382 10.1164/rccm.201308-1483ST PMC5803655

[5] Hansell DM (2013) Classification of diffuse lung diseases: why and how. Radiology 268: 628–640. 23970508 10.1148/radiol.13120908

[6] Trusheim MR, Berndt ER, Douglas FL (2007) Stratified medicine: strategic and economic implications of combining drugs and clinical biomarkers. Nature Reviews Drug Discovery 6: 287–293. 17380152 10.1038/nrd2251

[7] Cesuroglu T, van Ommen B, Malats N, Sudbrak R, Lehrach H, et al (2012) Public health perspective: from personalized medicine to personal health. Personalized Medicine 9: 115–119. 10.2217/pme.12.16 29758819

[8] Hayhurst MD, MacNee W, Flenley DC, Wright D, McLean A, et al (1984) Diagnosis of pulmonary emphysema by computerised tomography. Lancet 2: 320–322. 6146866 10.1016/s0140-6736(84)92689-8

[9] Lynch DA, Travis WD, Muller NL, Galvin JR, Hansell DM, et al (2005) Idiopathic interstitial pneumonias: CT features. Radiology 236: 10–21. 15987960 10.1148/radiol.2361031674

[10] Xaubet A, Agusti C, Luburich P, Roca J, Monton C, et al (1998) Pulmonary function tests and CT scan in the management of idiopathic pulmonary fibrosis. American journal of respiratory and critical care medicine 158: 431–436. 9700117 10.1164/ajrccm.158.2.9709008

[11] Akira M, Inoue Y, Kitaichi M, Yamamoto S, Arai T, et al (2009) Usual Interstitial Pneumonia and Nonspecific Interstitial Pneumonia with and without Concurrent Emphysema: Thin-Section CT Findings. Radiology 251: 271–279. 19221055 10.1148/radiol.2511080917

[12] Mooney JJ, Elicker BM, Urbania TH, Agarwal MR, Ryerson CJ, et al (2013) Radiographic Fibrosis Score Predicts Survival in Hypersensitivity Pneumonitis. Chest 144: 586–592. 23392130 10.1378/chest.12-2623

[13] Flaherty KR, Mumford JA, Murray S, Kazerooni EA, Gross BH, et al (2003) Prognostic implications of physiologic and radiographic changes in idiopathic interstitial pneumonia. American Journal of Respiratory and Critical Care Medicine 168: 543–548. 12773329 10.1164/rccm.200209-1112OC

[14] MacDonald SL, Rubens MB, Hansell DM, Copley SJ, Desai SR, et al (2001) Nonspecific Interstitial Pneumonia and Usual Interstitial Pneumonia: Comparative Appearances at and Diagnostic Accuracy of Thin-Section CT1. Radiology 221: 600–605. 11719652 10.1148/radiol.2213010158

[15] Bartholmai BJ, Raghunath S, Karwoski RA, Moua T, Rajagopalan S, et al (2013) Quantitative computed tomography imaging of interstitial lung diseases. J Thorac Imaging 28: 298–307. 23966094 10.1097/RTI.0b013e3182a21969 PMC3850512

[16] Maldonado F, Moua T, Rajagopalan S, Karwoski RA, Raghunath S, et al (2014) Automated quantification of radiological patterns predicts survival in idiopathic pulmonary fibrosis. European Respiratory Journal 43: 204–212. 23563264 10.1183/09031936.00071812

[17] Uppaluri R, Hoffman EA, Sonka M, Hunninghake GW, McLennan G (1999) Interstitial lung disease - A quantitative study using the adaptive multiple feature method. American Journal of Respiratory and Critical Care Medicine 159: 519–525. 9927367 10.1164/ajrccm.159.2.9707145

[18] Xu Y, Sonka M, McLennan G, Guo J, Hoffman EA (2006) MDCT-based 3-D texture classification of emphysema and early smoking related lung pathologies. Medical Imaging, IEEE Transactions on 25: 464–475. 10.1109/TMI.2006.870889 16608061

[19] Castaldi PJ, San Jose Estepar R, Mendoza CS, Hersh CP, Laird N, et al (2013) Distinct quantitative computed tomography emphysema patterns are associated with physiology and function in smokers. Am J Respir Crit Care Med 188: 1083–1090. 23980521 10.1164/rccm.201305-0873OC PMC3863741

[20] Kim SS, Seo JB, Lee HY, Nevrekar DV, Forssen AV, et al (2013) Chronic obstructive pulmonary disease: lobe-based visual assessment of volumetric CT by Using standard images—comparison with quantitative CT and pulmonary function test in the COPDGene study. Radiology 266: 626–635. 23220894 10.1148/radiol.12120385 PMC3558873

[21] Gietema HA, Muller NL, Fauerbach PV, Sharma S, Edwards LD, et al (2011) Quantifying the extent of emphysema: factors associated with radiologists' estimations and quantitative indices of emphysema severity using the ECLIPSE cohort. Acad Radiol 18: 661–671. 21393027 10.1016/j.acra.2011.01.011

[22] Sullivan DC (2008) Imaging as a quantitative science. Radiology 248: 328–332. 18641239 10.1148/radiol.2482080242

[23] Celli BR, Cote CG, Marin JM, Casanova C, Montes de Oca M, et al (2004) The body-mass index, airflow obstruction, dyspnea, and exercise capacity index in chronic obstructive pulmonary disease. N Engl J Med 350: 1005–1012. 14999112 10.1056/NEJMoa021322

[24] Pauwels RA, Buist AS, Calverley PM, Jenkins CR, Hurd SS, et al (2001) Global strategy for the diagnosis, management, and prevention of chronic obstructive pulmonary disease. NHLBI/WHO Global Initiative for Chronic Obstructive Lung Disease (GOLD) Workshop summary. Am J Respir Crit Care Med 163: 1256–1276. 11316667 10.1164/ajrccm.163.5.2101039

[25] Jones PW, Quirk FH, Baveystock CM, Littlejohns P (1992) A self-complete measure of health status for chronic airflow limitation. The St. George's Respiratory Questionnaire. Am Rev Respir Dis 145: 1321–1327. 1595997 10.1164/ajrccm/145.6.1321

[26] Frey BJ, Dueck D (2007) Clustering by passing messages between data points. Science 315: 972–976. 17218491 10.1126/science.1136800

[27] CLARKE KR (1993) Non-parametric multivariate analyses of changes in community structure. Australian journal of ecology 18: 117–143.

[28] Ley B, Ryerson CJ, Vittinghoff E, Ryu JH, Tomassetti S, et al (2012) A multidimensional index and staging system for idiopathic pulmonary fibrosis. Ann Intern Med 156: 684–691. 22586007 10.7326/0003-4819-156-10-201205150-00004

[29] Vestbo J, Hurd SS, Agusti AG, Jones PW, Vogelmeier C, et al (2013) Global strategy for the diagnosis, management, and prevention of chronic obstructive pulmonary disease: GOLD executive summary. Am J Respir Crit Care Med 187: 347–365. 22878278 10.1164/rccm.201204-0596PP

[30] Raghunath S, Rajagopalan S, Karwoski RA, Larson AG, Bartholmai BJ, et al (2012) Detail-on-demand visualization for lean understanding of lung abnormalities. Stud Health Technol Inform 173: 362–368. 22357019

[31] Luppi F, Spagnolo P, Cerri S, Richeldi L (2012) The big clinical trials in idiopathic pulmonary fibrosis. Curr Opin Pulm Med 18: 428–432. 22759771 10.1097/MCP.0b013e3283567ff9

[32] Lederer DJ (2013) Clinical trials in idiopathic pulmonary fibrosis: a framework for moving forward. European Respiratory Journal 42: 1442–1448. 10.1183/09031936.00092713 24293416

[33] Pellegrino R, Viegi G, Brusasco V, Crapo RO, Burgos F, et al (2005) Interpretative strategies for lung function tests. Eur Respir J 26: 948–968. 16264058 10.1183/09031936.05.00035205

[34] Ryerson CJ, Hartman T, Elicker BM, Ley B, Lee JS, et al (2013) Clinical features and outcomes in combined pulmonary fibrosis and emphysema in idiopathic pulmonary fibrosis. Chest 144: 234–240. 23370641 10.1378/chest.12-2403

[35] Schuster DP (2007) The opportunities and challenges of developing imaging biomarkers to study lung function and disease. Am J Respir Crit Care Med 176: 224–230. 17478617 10.1164/rccm.200703-462PP

[36] Goldin JG (2013) Computed tomography as a biomarker in clinical trials imaging. Journal of thoracic imaging 28: 291–297. 23966093 10.1097/RTI.0b013e3182a1d93d

[37] Best AC, Meng J, Lynch AM, Bozic CM, Miller D, et al (2008) Idiopathic Pulmonary Fibrosis: Physiologic Tests, Quantitative CT Indexes, and CT Visual Scores as Predictors of Mortality1. Radiology 246: 935–940. 18235106 10.1148/radiol.2463062200

[38] Lynch DA, Godwin JD, Safrin S, Starko KM, Hormel P, et al (2005) High-resolution computed tomography in idiopathic pulmonary fibrosis: diagnosis and prognosis. American journal of respiratory and critical care medicine 172: 488–493. 15894598 10.1164/rccm.200412-1756OC

[39] Dirksen A, Macnee W (2013) The search for distinct and clinically useful phenotypes in chronic obstructive pulmonary disease. Am J Respir Crit Care Med 188: 1045–1046. 24180437 10.1164/rccm.201309-1649ED

