# Supplementary figures and images for: Quantitative Stratification of Diffuse Parenchymal Lung Diseases
Source: PLoS One. 2014 Mar 27;9(3):e93229. doi: 10.1371/journal.pone.0093229 (PMC3968138; doi:10.1371/journal.pone.0093229)

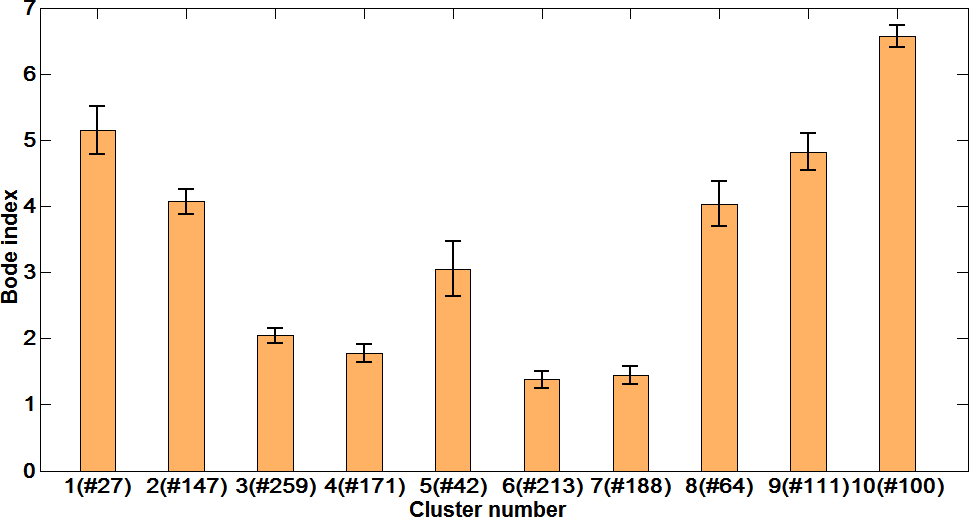

Supplement: Figure S1 — The mean distribution of the BODE indices across the ten clusters. The error bars represent standard error of mean. The BODE is generally defined for obstructive cluster and Figure 7D illustrates the distribution for clusters 6 through 10. The trend in BODE distribution across clusters resemble the SGRQ (Figure 7E). (TIF) [file pone.0093229.s001.tif]

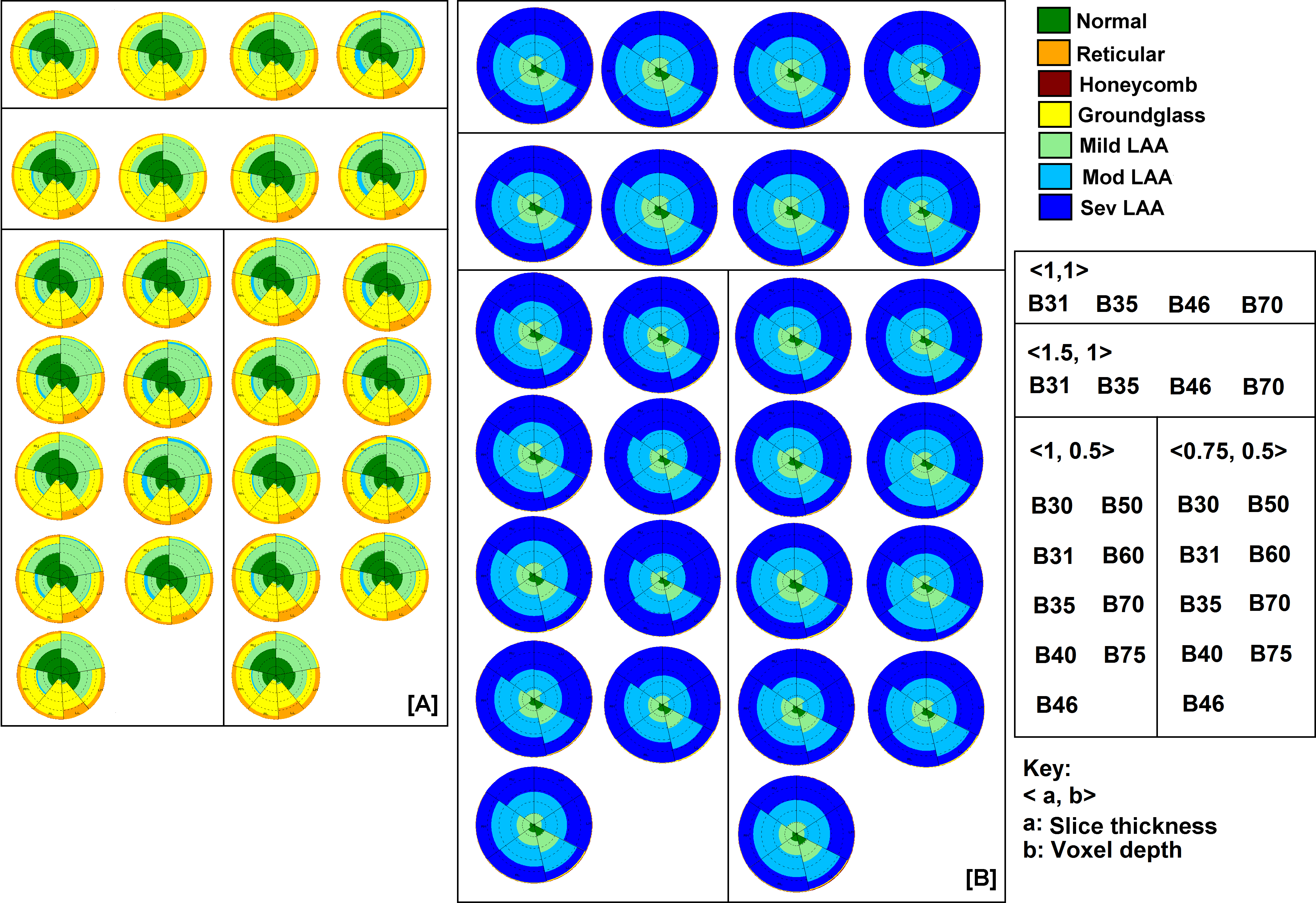

Supplement: Figure S2 — Glyph representations of multiple reconstructions of two LTRC patients. All the data reconstructions for both cases, (A) and (B) were consistently categorized into cluster 3 and cluster 10, respectively. (TIF) [file pone.0093229.s002.tif]
