# Supplementary material for: Quantitative Stratification of Diffuse Parenchymal Lung Diseases
Source: PLoS One. 2014 Mar 27;9(3):e93229. doi: 10.1371/journal.pone.0093229 (PMC3968138; doi:10.1371/journal.pone.0093229)
Supplement: Table S1 — Patient demographics and major diagnosis of LTRC cohort. (DOCX) [file pone.0093229.s003.docx]

**Supplementary Table**

Table S1: Patient demographics and major diagnosis of LTRC cohort.

| **Demographics** | **N = 1322** |
| --- | --- |
| **Age**  Years: median (range) | 64 (23-93) |
| **Gender** n (%)  ***Women*** | 647 (49%) |
| **Final Major Diagnosis** n (%) |  |
| **ILD** | 344 (26%) |
| **COPD** | 613 (46%) |
| **Control** | 133 (10%) |
| **Others** | 232 (18%) |
