# Supplementary material for: Quantitative Stratification of Diffuse Parenchymal Lung Diseases
Source: PLoS One. 2014 Mar 27;9(3):e93229. doi: 10.1371/journal.pone.0093229 (PMC3968138; doi:10.1371/journal.pone.0093229)
Supplement: File S1 — (DOCX) [file pone.0093229.s004.docx]

# Supplementary Information

## Stratification consistency across CT reconstructions

To understand the influence of CT reconstructions on CALIPER quantification and stratification, a detailed study was conducted using two representative cases. Raw CT data for two patients enrolled in LTRC and scanned using Siemens scanner were identified. From the raw data, multiple reconstructions were derived by changing slice thickness, slice overlap and reconstruction filter kernels. Reconstruction filter kernels specified as B<X><Y>f use linear or non-linear filtering operations during and/or post image reconstruction for smoothness or sharpness enhancements of image features. Lower values of X represent relatively smoother images and Y represents the specific filtering properties. For B31f, B35f, B46f and B70f kernels, volumes were reconstructed using <slice thickness, voxel depth> combinations of <1, 1> and <1.5, 1> mm. For B30f, B31f, B35f, B40f, B46f, B50f, B60f, B70f and B75f kernels, volumes were reconstructed using <1, 0.5> and <0.75, 0.5> mm. This exercise resulted in 26 reconstructions for each of the two cases.

The reconstructed volumes were processed using CALIPER to characterize the individual parenchymal voxels into one of the patterns. **Figure S2** shows glyph representations of the 26 reconstructions for the two cases. Case (A) and (B) represent respectively fibrotic and obstructive type of parenchymal abnormalities. The variations seen on the glyphs across the reconstruction settings are visually minimal. Furthermore, each reconstruction was categorized into one of the identified ten clusters. The categorization was performed by comparing the distribution (Equation 1 in the main paper) of radiological pattern in each reconstructed volume with each of the clusters. Nearest neighbor classification scheme was used to identify the reconstructed volumes with the most similar cluster. Cases (A) and (B) in **Figure S2** were respectively categorized into cluster 3 and 10. The categorization of all the reconstructions of the cases into the same cluster reflects the consistency in stratification.
